# Equity in initial health evaluation utilization among world trade center health program members enrolled during 2012–2022

**DOI:** 10.1186/s12913-025-13248-w

**Published:** 2025-08-05

**Authors:** Ruiling Liu, Michael O’Reilly, Sarah Rockhill, Lillian Fu, Kendra C. Smith, Emma Butturini, Albeliz Santiago-Colón, Rachael L. Shaw, Kevin Pressley, Geoffrey M. Calvert

**Affiliations:** 1 World Trade Center Health Program, National Institute for Occupational Safety and Health (NIOSH), Centers for Disease Control and Prevention, Atlanta, GA USA; 2 Rollins School of Public Health, Emory University, Atlanta, GA USA; 3 Geospatial Research, Analysis, and Services Program, Office of Innovation and Analytics, Agency for Toxic Substances and Disease Registry, Centers for Disease Control and Prevention, Atlanta, GA USA

**Keywords:** WTC Health Program, Initial health evaluations, Health service utilization, Healthcare equity

## Abstract

**Background:**

The World Trade Center (WTC) Health Program, a limited federal healthcare program, provides medical monitoring and treatment for WTC-related conditions to eligible Responders and Survivors of the 9/11 terrorist attacks. Free initial health evaluations (IHE) represent the first step towards the Program’s goal of providing equitable and timely member access to healthcare. This study aimed to evaluate equity in IHE utilization among Program members to inform the development of targeted interventions.

**Methods:**

This surveillance study used administrative and surveillance data collected from January 2012 through February 2024. It included Program members newly enrolled during 2012–2022 who completed an IHE or were alive for ≥ 1 year after enrollment. We conducted descriptive and multivariable logistic regression analyses. Outcomes of interest included timely IHE utilization (proportion of members completing an IHE within 6 months of enrollment) and any IHE utilization (proportion completing an IHE by February 2024). Factors of interest included member type, sex, age, race/ethnicity, preferred language, and urban/rural residence.

**Results:**

27,379 Responders and 30,679 Survivors were included. Responders were 89% male, 70% 45–64 years old at enrollment and 76% non-Hispanic White. Survivors were 54% male, 54% 45–64 years old at enrollment and 57% non-Hispanic White. Timely IHE utilization remained stable (~ 65%) among Responders, while for Survivors, it increased from 16% among those enrolled in 2017 to 68% in 2021. Timely IHE utilization was lower for younger members (enrolled < 45 years old vs. ≥ 65 years old, adjusted odds ratio [aOR] = 0.71, *p* < 0.001), rural residents, female Survivors (44% vs. 47% males, aOR = 0.87, *p* < 0.001), and Survivors who preferred non-English languages (39% vs. 46% who preferred English, aOR = 0.70, *p* < 0.001). Compared to non-Hispanic White members, non-Hispanic Black members had higher timely/any IHE utilization, while non-Hispanic Asian/Pacific Islander/Native Hawaiian and Hispanic Survivors had lower timely IHE utilization.

**Conclusions:**

This study highlights Program achievements (e.g. increased timely IHE utilization among Survivors over time and higher timely/any IHE utilization among non-Hispanic Black members compared to non-Hispanic White members) and gaps in providing equitable IHE services to its members. The Program can develop tailored strategies to further improve equity in IHE utilization (e.g. working with providers to adopt/expand flexible IHE scheduling and increase non-English language capacity).

**Supplementary Information:**

The online version contains supplementary material available at 10.1186/s12913-025-13248-w.

## Introduction

In January 2011, the James Zadroga 9/11 Health and Compensation Act of 2010 (Zadroga Act) was signed into law, creating the World Trade Center (WTC) Health Program (Program). The Program provides a limited benefit health plan for persons exposed to the terrorist attacks on September 11, 2001 (9/11) and its aftermath [1]. Beginning shortly after passage of the Zadroga Act, the Program provided monitoring and treatment for health conditions related to 9/11 exposures (WTC-related conditions), including cancer, aerodigestive disorders, and mental health conditions. As of June 30, 2024, there were over 130,000 Program members, including about 60,000 legacy members from predecessor programs and 70,000 members enrolled post-Zadroga. Membership has grown annually by approximately 7,500 since 2019; over 80,000 members are certified for at least one WTC-related condition and are eligible for related treatment benefits from the Program [2].

Program members are generally categorized as either Responders (who performed rescue, recovery, clean-up, or other related support services) or Survivors (who did not respond but were present in the New York disaster area on or after 9/11). High-level differences between these groups are described in Table 1. Once enrolled, members are assigned to one of 8 clinics in the New York Metropolitan Area or the Nationwide Provider Network (NPN) and are encouraged to complete an annual monitoring exam (AME, for Responders) or an initial health evaluation (IHE, for Survivors) to initiate services. The first AME or IHE (collectively IHE hereafter) is free to all members and is a comprehensive exam including: (1) detailed questionnaires evaluating 9/11 exposures, medical history, and mental health status; (2) blood and urine analyses; 4) spirometry; 5) chest radiography (when clinically indicated); and 6) electrocardiogram (for those ≥ 40 years old, when clinically indicated) [1]. Because the IHE is an essential prerequisite to initiating care and establishing a baseline for identifying and treating WTC-related conditions, the Program strongly encourages newly enrolled members to complete their IHE within 6 months of clinic/NPN assignment.

**Table 1:** Differences between world trade center (WTC) health program responders and survivors

	Responders	Survivors
Location(s) of exposure	New York, NY (south of Canal St in Manhattan and some other sites); Arlington, Virginia; Shanksville, Pennsylvania	New York Disaster Area (south of Houston St in Manhattan and any block in Brooklyn wholly or partially contained within a 1.5-mile radius of the former WTC site)
9/11 experience	Performed rescue, recovery, clean-up or related support services	Worked, lived, attended school, childcare, or adult care, or was otherwise present in the New York disaster area
WTC-related condition* symptoms	Not asked when submitting application for enrollment	Asked to report any symptoms when submitting enrollment application. If no symptoms reported, they are encouraged to delay enrollment until they have symptoms. If symptomatic, they are more likely to get certified after their one-time free initial health evaluation (IHE).
Eligibility for Monitoring	Free annual monitoring exams upon enrollment	One-time free IHE upon enrollment;
		Eligible for free annual monitoring exam after being certified for a WTC-related condition
Payment for treatment	WTC Health Program is the primary payer for medically necessary treatment of certified conditions	WTC Health Program is the payer of last resort for medically necessary treatment of certified conditions, after private or public insurance

*WTC-related condition: health conditions directly related to 9/11 exposures, including cancer, aerodigestive disorders, mental health conditions, acute traumatic injuries, and musculoskeletal disorders, as listed by the PHS Act [PHS Act, §§ 3312(a)(3) and 3322(b); 42 C.F.R. § 88.1]

The Program continuously pursues strategies to improve members’ timely access to IHEs. For example, in 2016, the Department of Justice’s September 11th Victim Compensation Fund (VCF), which was created to provide financial compensation to eligible individuals for physical harm or death caused by the 9/11 attacks and their aftermath [3], began requiring that living claimants be enrolled in and certified for treatment by the WTC Health Program prior to filing for compensation. This policy change led to a sharp increase in Program membership, especially among Survivor members, beginning in 2017 [4]. As a result, the Survivor clinics/NPN faced challenges in providing timely IHEs to newly enrolled members. In response to the rising demand, the Program established a new Survivor clinic in late 2018. Also, when in-person access to healthcare services was disrupted due to the Coronavirus disease 2019 (COVID-19), the Program expanded telemedicine services to include preventive care visits such as IHEs [5]. Some IHE components like spirometry, chest radiography and electrocardiogram could not be completed because of restricted in-person services.

Whether members have equitable IHE utilization has not been evaluated. However, prior research suggests that multiple factors are associated with disparities in healthcare access and utilization. Women are more likely than men to utilize primary care and preventative health services, while men are more likely to use emergency and inpatient services [6, 7]. Persistent disparities in health care access and use have been documented across racial and ethnic groups in the U.S., even after the implementation of healthcare reforms aimed at increasing access [6, 8, 9]. One study found Black and Hispanic/Latino individuals were more likely to encounter multiple barriers to timely healthcare, including lack of transportation or telephone assistance, long wait times, limited appointment availability, and inconvenient clinic hours [9]. Language barriers are also a well-documented contributor to healthcare inequalities [10–12]. In addition, rurality has been associated with poorer health outcomes across a range of conditions, often due to limited access to healthcare [13, 14].

The Program is committed to providing members with timely, equitable, and high-quality healthcare, and IHEs are the first step toward achieving that goal. This study aims to evaluate potential inequities in IHE utilization to inform Program intervention development.

## Methods

This surveillance study included WTC Health Program members who enrolled during 2012–2022. To allow at least one year for members to complete an IHE, we excluded members who died within one year of enrollment and hadn’t yet received their IHE.

### IHE utilization

An IHE was defined as a member’s first clinic screening or evaluation visit with a Program provider, identified through Program-paid medical claims processed as of February 2024. A detailed description of our data extraction approach is included in the appendix (eTable1). We calculated the time elapsed from members’ enrollment date to their IHE date and evaluated two outcomes of interest: (1) timely IHE utilization: the proportion of total enrolled members who received an IHE within six months of enrollment; and (2) any IHE utilization: the proportion of total enrolled members who received any IHE prior to February 2024.

### Variables

We used administrative data collected from January 2012 through February 2024 to obtain member-level variables, including member type, sex, enrollment age, race/ethnicity, language preference for routine communication, urban/rural residence, first assigned clinic, enrollment year, and any clinic transfer prior to the IHE.

Members’ race/ethnicity are collected via self-report mostly at IHEs and/or subsequent AMEs. We used members’ most recent self-reported race/ethnicity. If the value was reported as ‘other’, ‘unknown’, or missing, we used their most recent historical self-reported value that was not ‘other’, ‘unknown’, or missing. Approximately 38% of the study population never reported race/ethnicity information. For these members, we applied the validated modified Bayesian improved first name/surname geocoding (mBIFSG) method, using members’ first name, surname, and most recent residential address to generate probabilities for six exclusive race/ethnicity groups: Hispanic (regardless of race), American Indian or Alaska Native (AI/AN), Asian/Pacific Islander/Native Hawaiian (AAPI), Black, multiracial, and White [15, 16]. A brief mBIFSG description and imputed values are included in the appendix (eTable1). We combined self-reported race/ethnicity and imputed data and assigned a probability value of 1 to members with self-reported race/ethnicity. All analyses involving race/ethnicity were weighted using these probabilities.

To determine urban/rural status, members’ residential zip codes from administrative data were mapped to the 2010 Rural-Urban Commuting Area (RUCA) zip code file, which is the most recent version released by the U.S. Department of Agriculture [17]. Members’ residential zip code at the time closest to their IHE date (+/- one year) were used if available, otherwise, their most recent residential zip code from the Program’s administrative data was used. RUCA codes classify U.S. census tracts using measures of population density, urbanization, and daily commuting. Members were categorized as urban residents if their zip code was in a metropolitan area or an area with 30–50% of commuter traffic flowing to an urban area [18].

### Data analyses

We described IHE utilization by various member characteristics, including enrollment age, sex, and race/ethnicity. Multivariable logistic regression models were used to estimate adjusted odds ratios (aORs) for timely IHE utilization and any IHE utilization by sex, enrollment age, race/ethnicity, language preference, and urban/rural residence. Each model was adjusted for clinic transfers prior to IHE, enrollment year, and first assigned clinic/NPN. A *p*-value < 0.05 from the regression models was used to indicate statistically significant differences between comparison groups. Separate analyses were conducted for Responders and Survivors due to important differences between these groups. Because a new Survivor clinic was added in 2018 and IHE claims data for members enrolled after 2021 might be incomplete due to process lag, we conducted sensitivity analyses by including only members newly enrolled in 2019–2021. Results related to members’ first assigned clinic are not presented due to contractual restrictions.

Geocoding and all data analyses were conducted using ArcGIS Pro version 2.9 (Environmental Systems Research Institute) and SAS version 9.4 (SAS Institute).

### Human subjects protection

This activity was reviewed by CDC, deemed not research, and was conducted consistent with applicable federal law and CDC policy.^1^

## Results

We included 58,058 members who newly enrolled in the Program during 2012–2022, of whom 55% received a timely IHE and 85% received any IHE.

### Responder members

#### General characteristics (Table 2)

**Table 2:** Characteristics and IHE utilization as of Feb 2024 of WTC Health Program members newly enrolled during 2012–2022

	Responders						Survivors					
	All^3^		With timely IHE^4^		With any IHE^5^		All^3^		With timely IHE^4^		With any IHE^5^	
	*N*	Col %	*N*	Row %	*N*	Row %	*N*	Col %	*N*	Row %	*N*	Row %
All	27,379	100	17,816	65	24,566	90	30,679	100	14,080	46	24,660	80
Sex												
Female	3,124	11	1,974	63	2,775	89	14,036	46	6,199	44	11,222	80
Male	24,255	89	15,842	65	21,791	90	16,643	54	7,881	47	13,438	81
Age at enrollment												
< 45 years	4,022	15	2,496	62	3,708	92	3,469	11	1,283	37	2,475	71
45–64 years	19,194	70	12,558	65	17,237	90	16,665	54	7,609	46	13,634	82
≥ 65 years	4,163	15	2,762	66	3,621	87	10,545	34	5,188	49	8,551	81
Race/ethnicity^1^												
White	20,858	76	13,310	64	18,601	89	17,592	57	8,080	46	14,145	80
AI/AN	60	0	36	60	51	85	56	1	28	50	46	82
AAPI	528	2	384	73	486	92	3,401	11	1,473	43	2,531	74
Black	2,426	9	1,713	71	2,224	92	5,216	17	2,589	50	4,387	84
Multiracial/other	546	2	326	60	481	88	993	3	427	43	847	85
Hispanic	2,961	11	2,047	69	2,723	92	3,421	11	1,483	43	2,704	79
Preferred language												
English	27,189	99	17,685	65	24,392	90	29,752	97	13,723	46	24,004	81
Other	190	1	131	69	174	92	927	3	357	39	656	71
Rural/Urban^2^												
Rural	1,269	5	486	38	1,005	79	391	1	132	34	293	75
Urban	26,049	95	17,284	66	23,505	90	30,249	99	13,928	46	24,337	80
NA	61	0	46	75	56	92	39	0	20	51	30	77
With clinic transfer before IHE												
Yes	2,360	9	1,361	58	2,184	93	3,692	12	570	15	2,912	79
No	25,019	91	16,455	66	22,382	89	26,987	88	13,510	50	21,748	81

*WTC:* World Trade Center

^1^Including both imputed and self-reported data. These groups are mutually exclusive, i.e. all groups are non-Hispanic except the Hispanic group. AI/AN: American Indian/Alaskan Native; AAPI: Asian Americans/Pacific Islander/Native Hawaiian

^2^ Based on members’ residential zip code at time of linked surveys if available or most recent residential zip codes; Members were categorized as urban residents if their zip codes were in a Metropolitan area or an area with 30–50% of commuter traffic flowing to an urban area, using the most recent 2010 Rural-Urban Commuting Area (RUCA) zip code file released by U.S Department of Agriculture; otherwise they were classified as rural residents unless their zip code could not be geocoded (P.O. Box zip codes, military zip codes or outside of U.S. zip codes), which were coded as NA

^3^The percentages under ‘All’ are all column proportions, other percentages are row proportions

^4^Completed an IHE within 6 months after enrollment

^5^Completed an IHE as of February 2024 based on claims data

Responder members (*n* = 27,379) were predominantly male (89%), enrolled at ages 45–64 years (70%), preferred English (99%), and lived in urban areas (95%). Most Responders (65%) received a timely IHE, while 10% had no IHE recorded.

#### Enrollment and IHE utilization trends (Fig. 1)

New Responder enrollees increased substantially during 2017–2019, then decreased in 2020 to levels like those seen in 2013–2016. Timely IHE utilization remained relatively stable over time, with a slight dip among those enrolled in 2019. Any IHE utilization was generally constant, with a modest decrease among those enrolled in 2021 and 2022. No consistent differences in IHE utilization were observed by sex or age across enrollment years. However, AAPI, Black, and Hispanic Responders had similar or slightly higher IHE utilization than White Responders across enrollment years.

**Fig. 1:**
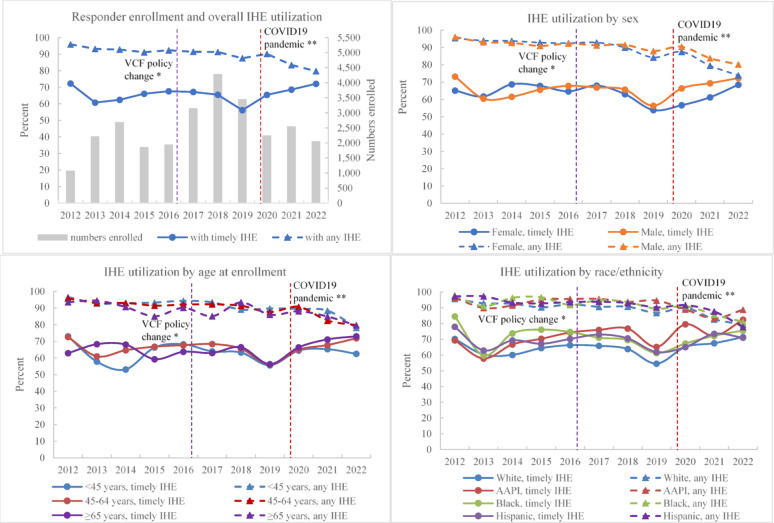
WTC Health Program Responder members: trends of enrollment and IHE utilization across enrollment years, 2012–2022.* WTC:* World Trade Center.* IHE:* initial health evaluation;* AAPI:* Asian/Pacific Islander/Native Hawaiian. Timely IHE: Received an IHE within 6 months of enrollment. Any IHE: Received an IHE anytime as of February 2024. Timely IHE utilization: proportion of members enrolled in a measurement year who received a timely IHE. Any IHE utilization: proportion of members enrolled in a measurement year who ever received an IHE as of February 2024. *In 2016, the Department of Justice’s September 11th Victim Compensation Fund (VCF) started to require claimants to be certified by the Program. **Coronavirus disease 2019 (COVID-19) pandemic started in the United States in 2020. These two events are noted for context, although analyzing the impact of these specific events is beyond the scope of the paper.

#### IHE utilization rate and adjusted odds ratios by different groups (Table 3)

**Table 3:** IHE utilization rates and adjusted odd ratios among WTC Health Program members newly enrolled during 2012–2022

Categories	Responders							Survivors						
	Total N	With timely IHE^3^			With any IHE^4^			Total N	With timely IHE^3^			With any IHE^4^		
		Row %	aOR (95%CI)	p value	Row %	aOR (95%CI)	p value		Row %	aOR (95%CI)	p value	Row %	aOR (95%CI)	p value
All	27,379	65			90			30,679	46			80		
Sex														
Female	3,124	63	0.93 (0.86, 1.02)	0.11	89	0.93 (0.82, 1.05)	0.23	14,036	44	**0.87 (0.82**,** 0.91)**	**< 0.001**	80	**0.92 (0.87**,** 0.98)**	**0.01**
Male	24,255	65	Reference		90	Reference		16,643	47	Reference		81	Reference	
Age														
< 45 years	4,022	62	**0.71 (0.64**,** 0.79)**	**< 0.001**	92	1.01 (0.86, 1.18)	0.94	3,469	37	**0.71 (0.65**,** 0.78)**	**< 0.001**	71	**0.60 (0.54**,** 0.66)**	**< 0.001**
45–64 years	19,194	65	0.93 (0.86, 1.00)	0.06	90	1.06 (0.95, 1.18)	0.28	16,665	46	0.97 (0.92, 1.02)	0.27	82	**1.08 (1.01**,** 1.15)**	**0.02**
≥ 65 years	4,163	66	Reference		87	Reference		10,545	49	Reference		81	Reference	
Race/Ethnicity^1^														
White	20,858	64	Reference		89	Reference		17,592	46	Reference		80	Reference	
AIAN	60	60	0.96 (0.58, 1.59)	0.87	85	0.72 (0.39, 1.36)	0.32	56	50	1.38 (0.84, 2.24)	0.20	82	1.06 (0.66, 1.70)	0.81
AAPI	528	73	1.05 (0.89, 1.23)	0.60	92	1.02 (0.81, 1.29)	0.86	3,401	43	**0.83 (0.76**,** 0.90)**	**< 0.001**	74	**0.70 (0.64**,** 0.77)**	**< 0.001**
Black	2,426	71	**1.23 (1.12**,** 1.35)**	**< 0.001**	92	**1.29 (1.13**,** 1.48)**	**< 0.001**	5,216	50	**1.10 (1.03**,** 1.18)**	**0.004**	84	**1.21 (1.12**,** 1.31)**	**< 0.001**
Multiracial/other	546	60	0.96 (0.81, 1.13)	0.60	88	1.00 (0.79, 1.25)	0.97	993	43	1.03 (0.90, 1.18)	0.67	85	**1.53 (1.31**,** 1.78)**	**< 0.001**
Hispanic	2,961	69	1.08 (0.99, 1.18)	0.08	92	**1.17 (1.03**,** 1.34)**	**0.02**	3,421	43	**0.92 (0.85**,** 0.99)**	**0.03**	79	0.96 (0.88, 1.05)	0.41
Preferred language														
English	27,189	65	Reference		90	Reference		29,752	46	Reference		81	Reference	
Other	190	69	0.75 (0.54, 1.03)	0.08	92	**0.57 (0.34**,** 0.97)**	**0.04**	927	39	**0.70 (0.60**,** 0.82)**	**< 0.001**	71	**0.66 (0.56**,** 0.77)**	**< 0.001**
Rural/Urban^2^														
Rural	1,269	38	0.91 (0.80, 1.03)	0.13	79	0.89 (0.77, 1.03)	0.13	391	34	**0.78 (0.63**,** 0.98)**	**0.04**	75	0.85 (0.67, 1.09)	0.20
Urban	26,049	66	Reference		90	Reference		30,249	46	Reference		80	Reference	
NA	61	75	1.50 (0.84, 2.71)	0.17	92	0.90 (0.38, 2.13)	0.80	39	51	1.37 (0.71, 2.66)	0.347	77	1.02 (0.48, 2.17)	0.95
With clinic transfer before IHE														
Yes	2,360	58	**0.75 (0.67**,** 0.84)**	**< 0.001**	93	**1.60 (1.34**,** 1.92)**	**< 0.001**	3,692	15	**0.22 (0.20**,** 0.24)**	**< 0.001**	79	**0.70 (0.63**,** 0.77)**	**< 0.001**
No	25,019	66	Reference		89	Reference		26,987	50	Reference		81	Reference	

*WTC:* World Trade Center.* IHE:* initial health evaluation.* aOR:* adjusted odd ratio;* CI:* confidence interval

aORs, 95% CIs and p values were estimated by multivariable logistic regression. Each logistic regression model included all variables listed in the first column, plus enrollment year and first clinic assigned (results not shown in the table)

Estimates that were statistically significant are bolded

^1^Including both imputed and self-reported data. These groups are mutually exclusive, i.e. all groups are non-Hispanic except the Hispanic group. AI/AN: American Indian/Alaskan Native; AAPI: Asian Americans/Pacific Islander/Native Hawaiian.

^2^Based on members’ residential zip code at time of linked surveys if available or most recent residential zip codes; Members were categorized as urban residents if their zip codes were in a Metropolitan area or an area with 30–50% of commuter traffic flowing to an urban area, using the most recent 2010 Rural-Urban Commuting Area (RUCA) zip code file released by U.S Department of Agriculture; otherwise they were classified as rural residents unless their zip code could not be geocoded (P.O. Box zip codes, military zip codes or outside of U.S. zip codes), which were coded as NA

^3^Completed an IHE within 6 months after enrollment

^4^Completed an IHE as of February, 2024 based on claims data

Timely IHE utilization rates and adjusted odds were lower for Responders aged < 45 years at enrollment (62% vs. 66% who were ≥ 65 years, aOR = 0.71, *p* < 0.001) and among those who transferred clinics before receiving IHEs (58% vs. 66% who didn’t transfer clinics, aOR = 0.75, *p* < 0.001). Although not statistically significant, rural Responders had lower rates and adjusted odds of receiving both timely and any IHEs compared to urban Responders. Responders who transferred clinics before receiving an IHE had a higher rate of any IHE (93%) and higher adjusted odds (aOR = 1.60, *p* < 0.001) compared to those without a clinic transfer (89%). Black, AAPI, and Hispanic Responders had slightly higher rates and adjusted odds of receiving timely and any IHEs than White Responders, though the findings were statistically significant for Black Responders only. IHE utilization by Responders was similar across sex and preferred language.

### Survivor members

#### General characteristics (Table 2)

Survivor members (*n* = 30,679) were 54% male, 54% enrolled at ages 45–64, 57% were White, and 97% preferred English. Overall, 46% of Survivors received a timely IHE, while 20% had not yet received one by study end.

#### Enrollment and IHE utilization trends (Fig. 2)

New Survivor enrollees increased greatly since 2017, with a dip in 2020. Timely IHE utilization decreased substantially among Survivors enrolled in 2017, then increased among those enrolled in every subsequent year except 2022. Any IHE utilization has remained stable among Survivors enrolled since 2018 except in 2022. Females had similar or slightly lower timely IHE utilization than males in most years, except among those enrolled in 2019. However, females’ any IHE utilization was comparable to that of males across enrollment years. Since 2015, Survivors aged < 45 years at enrollment had consistently lower timely IHE or any IHE utilization than older groups. AAPI Survivors consistently had lower or similar timely IHE utilization relative to White Survivors for all enrollment years except 2019, and consistently lower any IHE utilization than other groups except in 2022. Black Survivors consistently had higher timely IHE and any IHE utilization than White Survivors since 2014.

**Fig. 2:**
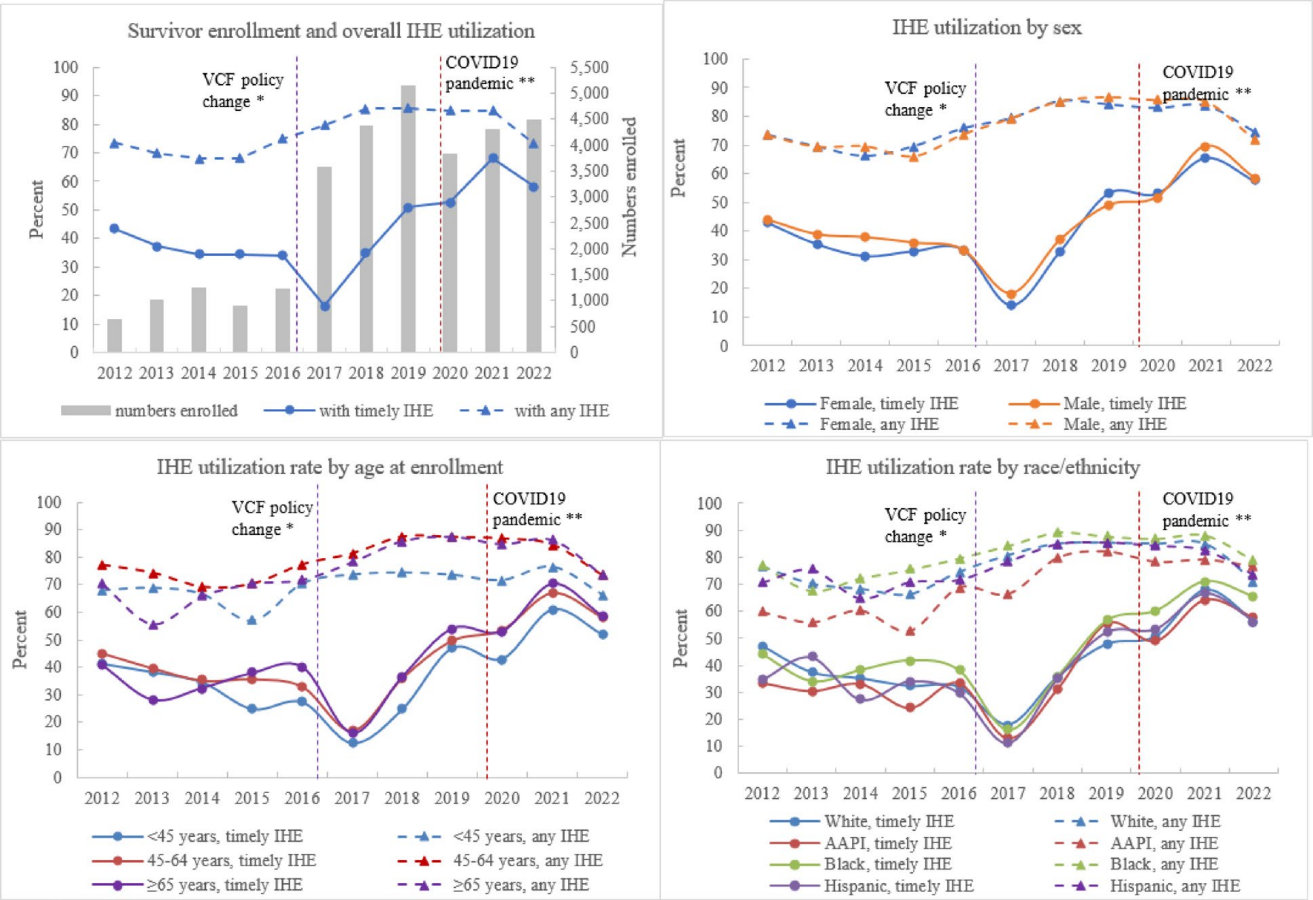
WTC Health Program survivor members: trends of enrollment and IHE utilization across enrollment years, 2012–2022.* WTC:* World Trade Center.* IHE:* initial health evaluation;* AAPI:* Asian/Pacific Islander/Native Hawaiian. Timely IHE utilization: proportion of members enrolled in a measurement year who received a timely IHE. Any IHE utilization: proportion of members enrolled in a measurement year who ever received an IHE as of February 2024. *In 2016, the Department of Justice’s September 11th Victim Compensation Fund (VCF) started to require claimants to be certified by the Program. **Coronavirus disease 2019 (COVID-19) pandemic started in the United States in 2020. These two events are noted for context, although analyzing the impact of these specific events is beyond the scope of the paper

#### IHE utilization rate and adjusted odds ratios by different groups (Table 3)

Timely IHE utilization rate and adjusted odds were lower among females (44% vs. 47% for males, aOR = 0.87, *p* < 0.001), individuals who enrolled when < 45 years old (37% vs. 49% among those aged ≥ 65 years old, aOR = 0.71, *p* < 0.001), those preferring non-English languages (39% vs. 46% for those preferring English, aOR = 0.70, *p* < 0.001), those who lived in rural areas (34% vs. 46% for urban residents, aOR = 0.78, *p* = 0.04), and individuals who transferred clinics before receiving their IHE (15% vs. 50% among those who didn’t transfer, aOR = 0.22, *p* < 0.001). Compared to White Survivors, Black Survivors were more likely to receive a timely IHE (50% vs. 46%, aOR = 1.10, *p* = 0.004), while AAPI (43%, aOR = 0.83, *p* < 0.001) and Hispanic Survivors (43%, aOR = 0.92, *p* = 0.03) were less likely. Similar patterns of disparities were observed for any IHEs, except there were no differences for female or Hispanic Survivors.

Sensitivity analyses showed similar findings when limited to members who enrolled during 2019–2021 (Appendix eTable 2).

## Discussion

This is the first study to evaluate equity in healthcare utilization among both Responders and Survivors of the WTC Health Program. IHEs are important as they are the first step to understanding member healthcare needs, identifying conditions eligible for certification, and determining member eligibility for additional Program-provided medical services. About 46% of Responders and 69% of Survivors were certified for a WTC-related condition within one year of their IHE (data not shown). Equitable access to a timely IHE is essential for the Program to subsequently provide members with equitable, timely, and necessary healthcare for WTC-related conditions. We found differences in timely IHE utilization among members, particularly among Survivors enrolled in different years, and identified disparities in IHE utilization for certain groups.

The Program has worked with clinics/NPN to improve members’ IHE utilization, with intensified efforts in recent years. These efforts include working directly with members and clinics to resolve issues related to provider availability, expanding office hours, and prioritizing access to care based on member acuity. Clinics/NPN are also required to assist members in locating and utilizing available social benefits, including transportation to the IHE. Overall, 85% of newly enrolled members received any IHE, and 55% received a timely IHE. Notably, timely IHE utilization among survivors increased significantly, from 16% among those enrolled in 2017 to 68% among those enrolled in 2021.

Timely IHE utilization among Survivors dropped substantially in 2017. This is likely due to a sharp increase in Survivor enrollment following a new VCF policy that required claimants to be enrolled in and certified for treatment by the Program prior to filing for compensation. The Program added a new Survivor clinic in 2018 to meet the increasing demand, and Survivors’ timely IHE utilization increased greatly from 2018 onward. A U.S. national study showed that wellness visits and preventive screenings in 2021 had not returned to pre-pandemic levels, with Asian adults experiencing the largest relative decrease within race/ethnicity groups [8]. However, trend analyses in our study showed members’ timely IHE utilization had increased notably since the onset of the pandemic across all sex, age, and race/ethnicity groups. This may be due to the Program’s expansion of telemedicine to various services, including IHEs [5], which made IHEs more accessible than in-person visits.

Our study showed that females had slightly lower IHE utilization compared to males, especially among Survivors. Previous studies have shown that females are more likely than males to utilize preventive health services [7, 19, 20], while others suggest they may be less likely to receive preventive services that are not sex-specific, such as colonoscopy and cholesterol screening [21–23]. Despite generally higher access and utilization, women report greater barriers to care, including non-financial obstacles, like time constraints from family and work obligations [24]. These barriers, along with the non-sex-specific nature of IHEs, may explain lower utilization among female members.

We found that members enrolled at ages < 45 years have lower timely IHE utilization compared to those aged ≥ 65 years, who often no longer work [25]. A previous study showed that younger working populations face greater accommodation barriers to timely care than older populations [24]. To improve members’ timely IHEs, clinics/NPN can expand clinic hours by, for example, providing evening or weekend appointments to accommodate working members. Other potential interventions include splitting the IHE into multiple parts, allowing some components to be completed prior to the in-person evaluation to minimize time spent in the clinic, or continuing to offer IHEs via telehealth. These strategies may better accommodate many individuals, including women and younger populations. It is also possible that younger adults, due to their better health status compared to older adults, have fewer healthcare demands [26] and may feel less motivated to complete a timely IHE to access Program medical benefits.

Both Black Responders and Survivors had higher IHE utilization than White members. This finding differs from the existing literature, which indicates that minority populations are more likely to face barriers to timely healthcare [9] and have lower healthcare utilization due to structural racism [27, 28]. Over the past decades, Black Americans have consistently had higher uninsured rates than White Americans, and about half of Black adults report worrying about medical bills [29]. As the WTC Health Program provides no cost monitoring and treatment services for WTC-related health conditions to all qualified members, the racial disparities in healthcare access outside of the Program may have motivated Program utilization, resulting in higher IHE participation among Black members.

Our study indicated a disparity in IHE utilization among AAPI and Hispanic Survivors; however, the root cause is unclear. One possible reason may be language barriers. AAPI or Hispanic Survivors have a higher likelihood of preferring a non-English language (21.7% and 41.5%, respectively) than White Survivors (0.1%) (data not presented). This racial/ethnic disparity in IHE utilization was not observed among Responders, possibly because their job requirements may have included English proficiency. Our findings also showed that Survivors who preferred a non-English language were much less likely to receive a timely IHE or any IHE. Language barriers can hinder or delay access to healthcare [10, 30], negatively impact the relationship between patients and healthcare professionals, and reduce quality of care, leading to poor health outcomes [31–33]. The Program offers printed resources in several non-English languages and provides translation and interpretation services across all call centers. Collaborating with clinics/NPN to expand other language proficiency efforts could further improve healthcare access for members who prefer non-English languages.

Although most Program members lived in urban areas, about 3% lived in rural regions. Consistent with the literature, members living in rural areas were less likely to receive timely IHEs or any IHE, likely due to provider shortages, transportation challenges, and geographic distance [13].

### Limitations

Our study has some limitations. First, IHE dates were identified via medical claims, and there is a lag between date of service and claims submission (the Program has a filing limit of 18 months). Therefore, IHEs completed after 2022 could be underestimated. However, this underestimate is likely non-differential across all members; sensitivity analyses including only members enrolled in 2019–2021 showed no significant deviations. Second, there was a large proportion of members missing self-reported race/ethnicity data and this missingness is associated with IHE utilization, as race/ethnicity information was mostly collected during the IHE then updated in follow-up annual exams. Those who did not have an IHE tended to have a higher rate of missing race/ethnicity. As such, to examine equity of IHE utilization by different race/ethnicity groups, we had to impute race/ethnicity for 38% of the study population. This might cause misclassification, especially for AI/AN and multiracial members. For this reason and due to the small numbers in the AI/AN and multiracial populations, we were unable to study IHE utilization in these groups. However, imputation for other race/ethnicity groups had high prediction accuracy in our study and others [15, 16]. Third, survey data were collected by different Program contractors and had varying levels of completeness on certain data elements such as member and household income, education, working status and occupation at time of survey, other health insurance and/or access to healthcare outside of the Program, and distance to IHE providers. As a result, our study was unable to analyze or adjust for these socioeconomic factors nor members’ geographic access to IHE services, which may bias the associations reported in this study. Fourth, members may have moved post-enrollment but before their IHE. If their address was not captured at the time of the IHE, we relied on their most recent residential address. This could have led to potential urban/rural misclassification, particularly among members who had not received a timely or any IHE. Additionally, the most recent RUCA data available is for 2010, so any subsequent changes in the urban/rural status at the zip code level (e.g., post-2010 urban sprawl) could have introduced further misclassification. Lastly, for some factors, certain subgroups had only a small proportion of Responders or Survivors, such as members who preferred a non-English language or lived in rural areas, limiting the statistical power to detect differences when compared to corresponding groups. Given the limitations discussed above, the related findings on IHE utilization disparity should be interpreted with caution.

## Conclusions

Timely IHE completion is essential for the WTC Health Program to provide equitable healthcare to its members. Using more than 10 years of administrative and survey data, this study highlighted Program achievements, such as increased timely IHE utilization among Survivors over the years and higher timely/any IHE utilization among Black members compared to White members, as well as gaps in equitable IHE service delivery. Strategies to further improve equitable IHE utilization among members may include working with clinics/NPNs to adopt or expand flexible IHE scheduling, increasing non-English language capacity, and facilitating transportation and other assistance for members with accessibility barriers.

## Electronic supplementary material

Below is the link to the electronic supplementary material.

Supplementary Material 1

## Data Availability

The datasets analyzed during the current study are not publicly available due to federal privacy restrictions, but deidentified data may be available from the World Trade Center Health Program through a valid and reasonable request. Please contact the corresponding author for requesting access to related data.

## Abbreviations

AAPI: Asian/Pacific Islander/Native Hawaiian
AI/AN: American Indian or Alaska Native
AME: Annual monitoring exam
aOR: Adjusted odds ratio
CDC: Centers for Disease Control and Prevention
IHE: Initial health evaluation
mBIFSG: Modified Bayesian improved first name/surname geocoding
NIOSH: National Institute for Occupational Safety and Health
NPN: Nationwide Provider Network
RUCA: Rural-urban commuting area
WTC: World Trade Center

## Footnotes

1 See e.g., 45 C.F.R. part 46.102(l)(2), 21 C.F.R. part 56; 42 U.S.C. § 241(d); 5 U.S.C. § 552a; 44 U.S.C. § 3501 et seq.
