# Supplementary material for: Equity in initial health evaluation utilization among world trade center health program members enrolled during 2012–2022
Source: BMC Health Serv Res. 2025 Aug 5;25:1024. doi: 10.1186/s12913-025-13248-w (PMC12323082; doi:10.1186/s12913-025-13248-w)
Supplement: Supplementary file 1 — Supplementary Material 1 [file 12913_2025_13248_MOESM1_ESM.docx]

**Equity in Initial Health Evaluation Utilization among World Trade Center Health Program Members Enrolled during 2012-2022**

**APPENDIX**

**Identify Initial Health Evaluation Date from Medical Claims Data**

In this study, initial health evaluation (IHE) service was defined as the first evaluation office visit with a provider of the World Trade Center (WTC) Health Program (hereby Program) and was extracted from medical claims paid by the Program as of February 2024. Medical claims data includes information collected via the CMS-1500 Claim Form for professional claims or the UB-04 Claim Form for institutional claims. Each claim line includes up to 12 International Classification of Disease, Ninth or Tenth depending on the year, Clinical Modification (ICD-9-CM or ICD-10-CM) codes, one Current Procedural Terminology (CPT) code for medical service and procedure performed [1] and the service date. The Program provides guidance for providers to bill for IHE or annual monitoring exam (AME) services. Briefly, for Survivors, providers should use the ICD-9-CM code V82.9 or ICD-10-CM code Z13.9 as the primary diagnosis to bill for IHE services and use the ICD-9-CM code V71.9 and ICD-10-CM code Z04.9 as the primary diagnosis to bill for AME services; for Responders, providers should use the ICD-9-CM code V71.9 and ICD-10-CM code Z04.9 as primary diagnosis for both IHE and AME services. Providers should also use a CPT code 99384, 99385, 99386 or 99205 for IHE related office visits and a CPT code 99394, 99395, 99396 or 99215 for AME related office visits.

Though rare, there were some situations when a member was reclassified to a different member type, or AME diagnosis or CPT codes were used for IHE services. As such, we first extracted all the claim lines with the ICD-CM codes and CPT codes related to IHE/AME listed above and identified the claim line with the earliest date of service by member as the IHE office visit claim. This earliest date of service was defined as the IHE date for the member.

**Race/ethnicity Imputation**

We first used self-reported race/ethnicity information collected from members’ most recent IHE/AME. If they reported ‘Other’, ‘Unknown’, ‘Prefer not to say’ or missing values, we then used historical IHE/AME data to impute members’ race/ethnicity. That is, we used the most recent historical self-reported race/ethnicity that was not ‘Other’, ‘Unknown’, ‘Prefer not to say’ or missing values based on data collected from previous IHE/AMEs if there were any. After this step, about 38% of the study population (n=22,102) was missing race/ethnicity information. For these members, we applied the modified Bayesian improved first name surname geocoding (mBIFSG) method developed by RAND to generate probabilities for six exclusive race/ethnicity groups: Hispanic (regardless of race); American Indian or Alaska Native (AI/AN); Asian American/Pacific Islander/Native Hawaiian (AAPI); Black; multiracial; and White. This approach has been validated and applied in other studies [2, 3].

Briefly, this imputation approach uses an individual’s first name, surname, residential address, and supporting files ( 167,000 last names from 2010 Census data and 4,250 first names from 2.5 million mortgage applications in 2007 and 2010, and 2020 Census block group racial and ethnic composition) to assign race and ethnicity probabilities [2, 3]. Individuals’ full residential address was geocoded to a census block group (12-digit FIPS code); if a full address was not available or could not be geocoded, then their 5-digit zip code was used to assign the member to a census tract (11-digit FIPS codes) based on which tract contained the geometric center of the zip code tabulation area. In cases where the full address or zip code was not available, their residential state was used and geocoded to 2-digit FIPS code. Among the individuals included for the imputation, 92.1% had full residential addressed geocoded, 7.6% had only 5-digit zip codes available and 0.3% had only states available. All geocoding was performed via SAS 9.4 (for full address or states) or ArcGIS Pro v2.9 (for zip codes). The output is 6 imputed probabilities for the 6 exclusive race/ethnicity groups summing to 1 for each member, rather than assigning a single race/ethnicity value to a member. For example, a member could have imputed values like 80% being White, 10% being Hispanic, 5% being multiracial, 3% being Black, 1% being AI/AN and 1% being AAPI. Support datasets and SAS codes for race/ethnicity imputation were provided by RAND.

We also imputed race/ethnicity for the 35,795 individuals with self-reported information for the purpose of evaluating this imputation approach. We estimated discrimination using the concordance statistic (C-statistic), which was derived from area under the curve (AUC) analysis by fitting separate logistic regression model for each race/ethnicity group. As a general rule, a C-statistic value of 0.5 means the model is no better at predicting an outcome compared to random chance, values over 0.7 indicate a good model, values over 0.8 indicate a strong model, and a value of 1 means a perfect prediction [4]. See eTable 1 for imputed and self-reported race/ethnicity values for the study population. The imputation for AAPI, White, Black and Hispanic people was strong or excellent, but not that good for AI/AN or multiracial individuals.

The imputed data were combined with self-reported race/ethnicity. Therefore, we grouped members with self-reported race/ethnicity into the same six exclusive groups, combining those reported ‘other’ or ‘multiracial/other’ with multiracial; we assigned a probability value of 1 to those with self-reported race/ethnicity. All analyses involving race/ethnicity were weighted with the probabilities. The population of self-reported AI/AN or multiracial/other in our study is too small, and imputation for these two groups is not reliable, thus our study was not able to examine disparities of access to IHE among these two race/ethnicity groups.

**Sensitivity Analysis**

Because a new Survivor clinic was added in 2018 and claims data for IHEs in 2023 might be incomplete due to process lag, sensitivity analyses were conducted by including members enrolled in 2019-2021 only. For each outcome (timely IHE access and any IHE access) for responders and survivors, respectively, a multivariable logistic regression model was run to examine potential inequities by sex, age at enrollment, language preference, urban/rural residency, adjusted for any clinic transfers prior to IHE, enrollment year, and first assigned clinics. See eTable2 for detailed results. Results related to members’ first assigned clinic were not presented due to contractual restrictions.

**eTable 1 Self-reported and imputed race/ethnicity for World Trade Center Health Program members newly enrolled during 2012-2022**

| Race/Ethnicity^1^ | All | | Source | | | | Concordance Statistic between self-report and imputation^4^ |
| --- | --- | --- | --- | --- | --- | --- | --- |
|  |  |  | Imputed^2^ | | Self-reported^3^ | |  |
|  | N | % | N | % | N | % |  |
| All | 58,058 | 100.00 | 22,102 | 100.00 | 35,956 | 100.00 |  |
| White | 38,450 | 66.23 | 15,279 | 69.13 | 23,171 | 64.44 | 0.82 |
| AI/AN | 117 | 0.20 | 34 | 0.15 | 83 | 0.23 | 0.70 |
| AAPI | 3,928 | 6.77 | 1,678 | 7.59 | 2,250 | 6.26 | 0.97 |
| Black | 7,642 | 13.16 | 2,503 | 11.32 | 5,139 | 14.29 | 0.95 |
| Multiracial/Other | 1,539 | 2.65 | 269 | 1.22 | 1,270 | 3.53 | 0.60 |
| Hispanic | 6,382 | 10.99 | 2,339 | 10.58 | 4,043 | 11.24 | 0.93 |

Notes: ^1^ These groups are mutually exclusive, i.e. all groups are non-Hispanic except the Hispanic group.

AI/AN: American Indian/Alaskan Native; AAPI: Asian/Pacific Islander/Native Hawaiian. ‘Other’ were self-reported only. As some clinic collected data on a combined category ‘Multiracial/Other’ while some other clinics collected ‘Multiracial’ and ‘Other’ separately, these two categories were combined to one category. ^2^ Based on members’ surname, first name and most recent residential addresses, using the modified Bayesian improved first name surname geocoding (mBIFSG) method developed by RAND. ^3^ Self-reported race/ethnicity were collected at members’ initial health evaluations or annual monitoring evaluations. ^4^ This was based on separate logistic regression model for each race/ethnicity group among individuals with self-reported race/ethnicity data.

**eTable 2 Multivariable logistic regression analyses on IHE Utilizations among World Trade Center Health Program members enrolled during 2019-2021**

|  | Responders | | | | | | | Survivors | | | | | | |
| --- | --- | --- | --- | --- | --- | --- | --- | --- | --- | --- | --- | --- | --- | --- |
|  | Total N | With timely IHE^3^ | | | With any IHE^4^ | | | Total N | With timely IHE^3^ | | | with any IHE^4^ | | |
| Categories |  | Row % | OR (95%CI) | *p* | Row % | OR (95%CI) | *p* |  | Row % | OR (95%CI) | *p* | Row % | OR (95%CI) | *p* |
| All | 8,208 | 63 |  |  | 87 |  |  | 13,278 | 57 |  |  | 85 |  |  |
| Sex |  |  |  |  |  |  |  |  |  |  |  |  |  |  |
| Female | 932 | 57 | 0.86 (0.74, 1.01) | 0.07 | 84 | 0.84 (0.69, 1.03) | 0.09 | 5,802 | 57 | **0.86 (0.8, 0.93)** | **<0.001** | 84 | **0.83 (0.75, 0.92)** | **<0.001** |
| Male | 7,276 | 63 | Reference |  | 87 | Reference |  | 7,476 | 57 | Reference |  | 86 | Reference |  |
| Age at enrollment |  |  |  |  |  |  |  |  |  |  |  |  |  |  |
| <45 years | 586 | 60 | **0.67 (0.53, 0.84)** | **<0.001** | 89 | 1.02 (0.74, 1.4) | 0.92 | 1,339 | 49 | **0.65 (0.57, 0.74)** | **<0.001** | 74 | **0.43 (0.37, 0.5)** | **<0.001** |
| 45-64 years | 5,908 | 62 | **0.88 (0.77, 1)** | **0.04** | 87 | 0.99 (0.84, 1.18) | 0.93 | 7,046 | 57 | 0.94 (0.87, 1.02) | 0.12 | 86 | 1.00 (0.89, 1.11) | 0.95 |
| ≥65 years | 1,714 | 65 | Reference |  | 86 | Reference |  | 4,893 | 60 | Reference |  | 86 | Reference |  |
| Race/ethnicity^1^ |  |  |  |  |  |  |  |  |  |  |  |  |  |  |
| White | 6,296 | 62 | Reference |  | 86 | Reference |  | 7,782 | 56 | Reference |  | 85 | Reference |  |
| AIAN | 12 | 69 | 2.56 (0.99, 6.63) | 0.05 | 80 | 0.83 (0.3, 2.31) | 0.73 | 19 | 57 | 0.91 (0.41, 2.05) | 0.83 | 80 | 0.67 (0.29, 1.54) | 0.35 |
| AAPI | 178 | 71 | 1.04 (0.79, 1.37) | 0.77 | 89 | 0.93 (0.65, 1.35) | 0.71 | 1,469 | 57 | **0.79 (0.70, 0.90)** | **<0.001** | 80 | **0.72 (0.62, 0.84)** | **<0.001** |
| Black | 763 | 67 | **1.24 (1.06, 1.46)** | **0.01** | 89 | **1.29 (1.05, 1.6)** | **0.0180** | 2,289 | 62 | **1.07 (0.97, 1.19)** | **0.153** | 88 | **1.2 (1.05, 1.37)** | **0.01** |
| Multiracial/other | 147 | 53 | 1.03 (0.77, 1.38) | 0.84 | 85 | 1.22 (0.84, 1.78) | 0.30 | 400 | 54 | 0.95 (0.77, 1.16) | 0.58 | 87 | **1.37 (1.06, 1.77)** | **0.02** |
| Hispanic | 812 | 66 | 1.14 (0.97, 1.33) | 0.11 | 90 | **1.3 (1.03, 1.63)** | **0.03** | 1,319 | 57 | 0.94 (0.83, 1.06) | 0.29 | 84 | 0.97 (0.83, 1.13) | 0.69 |
| Preferred language |  |  |  |  |  |  |  |  |  |  |  |  |  |  |
| English | 8,186 | 63 | Reference |  | 87 | Reference |  | 12,909 | 57 | Reference |  | 85 | Reference |  |
| Other | 22 | 59 | 0.53 (0.21, 1.34) | 0.18 | 73 | **0.19 (0.08, 0.46)** | **<0.001** | 369 | 55 | **0.67 (0.53, 0.86)** | **0.001** | 80 | 0.76 (0.56, 1.01) | 0.06 |
| Rural/Urban ^2^ |  |  |  |  |  |  |  |  |  |  |  |  |  |  |
| Rural | 306 | 32 | 0.86 (0.66, 1.12) | 0.27 | 70 | 0.8 (0.62, 1.04) | 0.10 | 160 | 41 | 0.80 (0.58, 1.11) | 0.18 | 79 | 0.73 (0.5, 1.08) | 0.12 |
| Urban | 7,890 | 64 | Reference |  | 88 | Reference |  | 13,103 | 57 | Reference |  | 85 | Reference |  |
| NA | 12 | 67 | 1.05 (0.33, 3.39) | 0.94 | 92 | 1.05 (0.16, 7.04) | 0.96 | 15 | 40 | 0.63 (0.22, 1.80) | 0.388 | 73 | 0.61 (0.19, 1.95) | 0.40 |
| With clinic transfer before IHE | |  |  |  |  |  |  |  |  |  |  |  |  |  |
| Yes | 766 | 59 | **0.54 (0.44, 0.66)** | **<0.001** | 92 | 1.31 (0.98, 1.75) | 0.07 | 307 | 34 | **0.31 (0.23, 0.41)** | **<0.001** | 76 | **0.54 (0.41, 0.72)** | **<0.001** |
| No | 7,442 | 63 | Reference |  | 87 | Reference |  | 12,971 | 58 | Reference |  | 85 | Reference |  |

Notes: Each logistic regression model included all variables listed in the first column, plus first clinic assigned and year of enrollment, which results were not shown in the table.

Estimates that were statistically significant were bolded.

IHE: initial health evaluation; OR: odd ratio; CI: confidence interval.

^1^ Including both imputed and self-reported data. These groups are mutually exclusive, i.e. all groups are non-Hispanic except the Hispanic group. AI/AN: American Indian/Alaskan Native; AAPI: Asian Americans/Pacific Islander/Native Hawaiian. ^2^ Based on members’ residential zip code at time of linked surveys if available or most recent residential zip codes; Members were categorized as urban residents if their zip codes were in a Metropolitan area or an area with 30-50% of commuter traffic flowing to an urban area, using the most recent 2010 Rural-Urban Commuting Area (RUCA) zip code file released by U.S Department of Agriculture; otherwise they were classified as rural residents unless their zip code could not be geocoded (P.O. Box zip codes, military zip codes or outside of U.S. zip codes), which were grouped to NA; ^3^ Completed an IHE within 6 months after enrollment; ^4^ Completed an IHE as of February, 2024 based on claims data.

**References**

1. **American Medical Association CPT Codes** [<https://www.ama-assn.org/topics/cpt-codes>]

2. Haas A, Elliott MN, Dembosky JW, Adams JL, Wilson-Frederick SM, Mallett JS, Gaillot S, Haffer SC, Haviland AM: **Imputation of race/ethnicity to enable measurement of HEDIS performance by race/ethnicity**. *Health Services Research* 2019, **54**(1):13-23.

3. Branham DK, Finegold K, Chen L, Sorbero M, Euller R, Elliott MN, Sommers BD: **Trends in Missing Race and Ethnicity Information After Imputation in HealthCare.gov Marketplace Enrollment Data, 2015-2021**. *JAMA Network Open* 2022, **5**(6):e2216715-e2216715.

4. Hosmer DW, and S. Lemeshow: **Applied Logistic Regression**, 2nd edn. New York: Wiley Interscience Publication; 2000.
